# “Caterpillar sign” in corpus callosum associated with curvilinear pericallosal lipoma in MRI: A case report

**DOI:** 10.1016/j.radcr.2024.02.058

**Published:** 2024-03-05

**Authors:** Kazutoshi Konomatsu, Yosuke Kakisaka, Shiho Sato, Takafumi Kubota, Temma Soga, Kazushi Ukishiro, Kazutaka Jin, Shunji Mugikura, Masashi Aoki, Nobukazu Nakasato

**Affiliations:** a Departments of Epileptology, Tohoku University Graduate School of Medicine, Sendai, Miyagi, Japan; b Departments of Neurology Tohoku University Graduate School of Medicine, Sendai, Miyagi, Japan; c Departments of Diagnostic Radiology, Tohoku University Graduate School of Medicine, Sendai, Miyagi, Japan; d Division of Image Statistics, Tohoku Medical Megabank Organization, Sendai, Miyagi, Japan

**Keywords:** Curvilinear pericallosal lipoma, Caterpillar sign, Brain magnetic resonance imaging, Computed tomographic venography, Epilepsy

## Abstract

Lipoma of the corpus callosum, also known as pericallosal lipoma, is a rare congenital brain abnormality associated with corpus callosum dysgenesis or agenesis. Two morphological types are described: tubulonodular and curvilinear, with the latter being mostly asymptomatic. We present the case of a 30-year-old woman with epilepsy, whose magnetic resonance imaging revealed a “caterpillar sign” in the corpus callosum associated with a curvilinear pericallosal lipoma. The “caterpillar sign” in the corpus callosum showed low signal intensity on magnetization prepared rapid acquisition with gradient echo, high signal on fluid-attenuated inversion recovery, and low on susceptibility-weighted imaging, possibly indicating abnormal blood vessels penetrating from the ventricle to the posterior callosal vein. We need to be conscious of this unusual finding, particularly when considering surgical intervention in the corpus callosum in cases of pericallosal lipoma, to avoid vascular complications.

## Introduction

Intracranial lipomas are rare fat containing congenital lesions representing less than 0.1% of all intracranial tumors [1]. Pericallosal lipomas constitute the most common variety of intracranial lipomas (40%-50%) and are found in all age groups without sex predilection [2]. There are 2 morphological types of pericallosal lipomas: curvilinear and tubulonodular [3]. The curvilinear type is usually posterior, thin, less than 1 cm in diameter, and asymptomatic. The tubulonodular type is usually anterior, round, or lobular; generally thicker than 2 cm in diameter; and is more frequently symptomatic. Pericallosal lipomas are typically discovered incidentally and grow very slowly [4]. Multiple "slits" in the corpus callosum, a morphology that may be akin to a "caterpillar", have rarely been observed in cases with pericallosal lipomas on brain magnetic resonance imaging (MRI) [5]. Here, we report the case of a patient with epilepsy whose brain MRI revealed “caterpillar sign” in corpus callosum associated with curvilinear pericallosal lipoma.

## Case report

A 30-year-old woman was referred to the emergency department of a general hospital due to a generalized convulsion. She had no remarkable medical history; however, her sister and grandmother had epilepsy. Neurological examinations and blood investigations were unremarkable. Brain MRI showed linear hyperintense lesion over the corpus callosum on T1-weighted and T2-weighted images. Because inferior sagittal sinus thrombosis was suspected, a contrast-enhanced computed tomographic (CT) venography was performed. It showed no abnormalities in the venous sinus; however, a low-density lesion without calcification at the pericallosal space was observed.

Subsequently, the patient experienced recurrent generalized convulsions monthly. Short-term scalp electroencephalography (EEG) showed no abnormalities. However, based on her familial medical history, epilepsy was suspected, and administration of levetiracetam was initiated. The patient was then referred to our hospital for further diagnosis and pathophysiological examination.

**Fig. 1:**
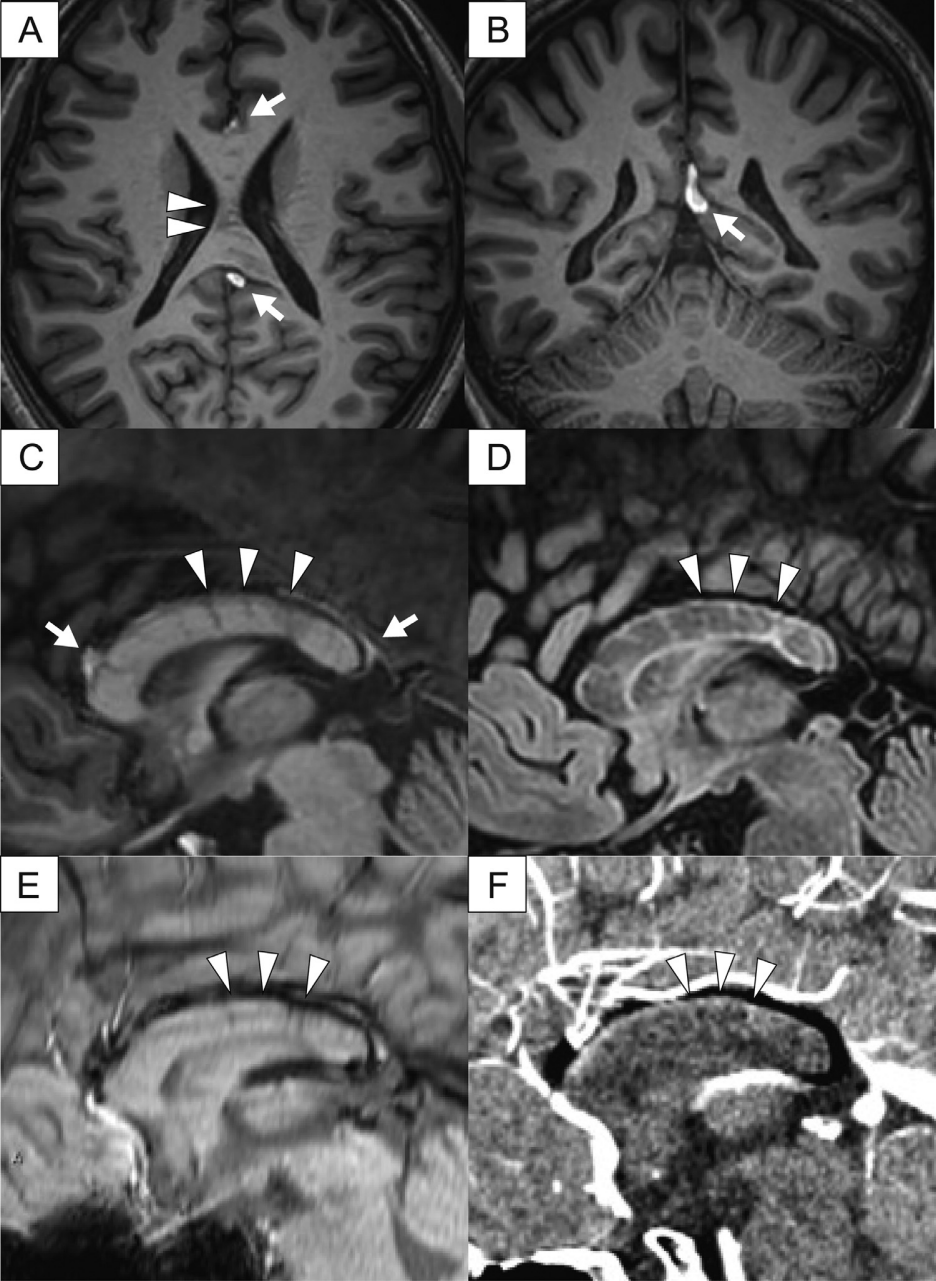
Imaging studies. Axial, coronal and sagittal magnetic resonance imaging of the brain shows a curvilinear hyperintense lesion along the somewhat hypoplastic corpus callosum on magnetization-prepared rapid acquisition with gradient echo (MPRAGE) (A, B, C), indicating a pericallosal lipoma of curvilinear type (arrow). Within the corpus callosum, “caterpillar sign” (arrowheads) are seen with low signal on MPRAGE (A, C), high signal on fluid-attenuated inversion recovery (D), low signal on susceptibility-weighted imaging (SWI) (E), and slightly contrast-enhanced on postcontrast computed tomography (F), respectively.

Neurological examination and blood investigations were unremarkable. Long-term video EEG monitoring (LTVEM) showed interictal spikes in the left frontal region. Additionally, one focal to bilateral tonic-clonic seizure was recorded. It began with forced eye deviation to the right, followed by right-side head turning, dystonic posturing of the right arm, and tonic-clonic convulsions. An ictal EEG showed onset in the left frontal region. Brain MRI showed a linear high signal intensity lesion over the corpus callosum on magnetization-prepared rapid acquisition with gradient echo (MP-RAGE) (Figs. 1 A-C, respectively) and fluid-attenuated inversion recovery (FLAIR) with hypoplasia of the corpus callosum (Fig. 1 D). Lesions containing multiple "slits," which may also be called the "caterpillar sign," were observed in the corpus callosum, with low signal intensity on MP-RAGE, strong signal on FLAIR, low signal on susceptibility-weighted imaging (SWI), and slightly contrasted in CT venography (Fig. 1 C-F, respectively). There were no findings of thrombosis in dural sinus or deep veins such as internal jugular vein, or vascular malformations such as arteriovenous malformation, or brain tumors other than pericallosal lipoma. Therefore, the lesions with multiple “slits” were speculated to be abnormal blood vessels penetrating the corpus callosum from the ventricle to the posterior callosal vein. The patient was eventually diagnosed with left frontal lobe epilepsy and levetiracetam was continued.

## Discussion

Our report presents the case of a patient with frontal lobe epilepsy with curvilinear pericallosal lipoma characterized by a unique pattern of multiple “slits” in corpus callosum on brain MRI, which might be called as “caterpillar sign”. Detailed evaluation of intracranial lipomas is crucial since they can frequently cause various pathological conditions, such as epilepsy and headaches, and to rule out more serious issues (such as venous thrombosis or malignant tumor) [6]. Additionally, our case has revealed the possible presence of an abnormal vascular pathology in the corpus callosum.

In our case, multiple “slits” in the corpus callosum were speculated as veins with perivascular space based on MRI findings, particularly the SWI sequence. SWI can sensitively detect veins because of their inherent deoxyhemoglobin content [7]. In this case, SWI showed striped lesions with low signal intensity, which were considered to be abnormal blood vessels penetrating the corpus callosum from the ventricle to the posterior callosal vein, based on their distribution. A similar finding was reported in a previous study on cranial lipoma, although observation of multiple “slits” in corpus callosum was not mentioned [5]. Given the aforementioned imaging and anatomical features, including the presence of a curvilinear pericallosal lipoma between the inferior sagittal sinus and the deep vein, we propose that this "slit" represents dilated collateral vessels.

Evaluation of the veins around pericallosal lipomas is important for several reasons, such as, venous infarction can be caused by injury when corpus callosotomy or biopsy of a lesion around corpus callosum is performed [8], [9], [10], [11]. The “caterpillar sign” of the corpus callosum shown here may be one of the unique features of curvilinear pericallosal lipoma and a coexisting anomaly of the corpus callosum, although it may simply be a speculation due to paucity of case reports [5]. Further accumulation of similar cases is required to elucidate detailed aspects of this condition.

In this case, the association between lipoma and epilepsy is unclear. Most symptomatic lipomas are tubulonodular and can cause epileptogenicity by compressing brain parenchyma. However, a previous report suggested that lipomas do not cause epilepsy [12]. Because 1) our case was a curvilinear type without compressing the brain parenchyma and 2) EEG abnormalities were observed in the left frontal region, we concluded that the lipoma was unrelated to epilepsy.

In conclusion, we report a case of “caterpillar sign” in the corpus callosum associated with curvilinear pericallosal lipoma. We need to be aware of this unusual finding, particularly when considering surgical intervention in the corpus callosum in situations of pericallosal lipoma, to minimize vascular complications during surgery.

## Patient consent

Written informed consent was obtained from the patient for publication of this case report and accompanying images.
